# B‐cell lymphoblastic lymphoma of the nictitating membrane as the first presenting sign in a 2‐year‐old Springer Spaniel

**DOI:** 10.1002/ccr3.1862

**Published:** 2018-10-12

**Authors:** Frederik Holm, Tommy Hardon, Erik Clasen‐Linde, Lauge Hjorth Mikkelsen, Steffen Heegaard

**Affiliations:** ^1^ Department of Pathology, Rigshospitalet University of Copenhagen Copenhagen Denmark; ^2^ Haslev Dyreklinik Haslev Denmark; ^3^ Department of Ophthalmology, Rigshospitalet‐Glostrup University of Copenhagen Copenhagen Denmark

**Keywords:** canine, immunohistochemistry, lymphoblastic, lymphoma, neoplasia, nictitating membrane

## Abstract

B‐cell lymphoblastic lymphoma is an aggressive malignant disease. Necropsy and microscopical examination revealed widespread disease with a high proliferation index. This is the first reported case of B‐cell lymphoblastic lymphoma presenting in the ocular region and only the second reported lymphoma of the nictitating membrane.

## 1 INTRODUCTION

A 2‐year‐and‐7‐month‐old, intact, female Springer Spaniel presented with a 1‐month history of a right‐sided nictitating membrane tumor. The tumor was mildly hyperemic and flabby. No other clinical signs were noticed, and the dog was in good general condition. After continual growth, the tumor was removed surgically. At 1‐month follow‐up, the general condition of the dog had deteriorated. A diagnosis of malignant lymphoma was reported, and the dog was euthanized. Necropsy revealed widespread disease, with tumors in the spleen, liver, and left ovary, as well as lymphadenopathy of several tonsillar, sternal, and intestinal lymph nodes. Microscopic and immunohistochemical examination showed diffuse infiltration of pleomorphic tumor cells of blast morphology in the nictitating membrane, spleen, liver, tonsils, and bone marrow. The tumor cells were positive for PAX5 and negative for CD3 and CD34. The Ki‐67 proliferation index of the nictitating membrane tumor was 90%, indicating very aggressive disease. Based on these findings, the final diagnosis was consistent with a B‐cell lymphoblastic lymphoma stage VB. B‐cell lymphoblastic lymphoma is less common than T‐cell lymphoblastic lymphoma in dogs. Lymphoblastic lymphomas are very aggressive, and widespread disease is common. To our knowledge, this is the first reported case of B‐cell lymphoblastic lymphoma presenting initially in the ocular region in a dog.

Lymphoid malignancies are common neoplasms of the canine family, with an incidence of 134/100 000 dogs,^1^ and account for 24% of all neoplasms.^2^ B‐cell lymphoblastic lymphoma (B‐LBL) is a precursor subtype of lymphoma that accounts for 1.8%‐2.4%^3^, ^4^ of all canine lymphomas.

Both B‐LBL and T‐cell lymphoblastic lymphoma (T‐LBL) in humans are characterized by small to medium‐sized blast cells with scant cytoplasm and a moderately condensed chromatin and inconspicuous nucleoli.^5^ However, the immunohistochemical profile is different in the B‐cell and T‐cell variants. In humans, tumor cells of B‐LBL are positive for terminal deoxynucleotidyl (TdT) and usually positive for CD19 and cytoplasmic CD79α. In most cases, the tumors cells are positive for CD10 and CD24, and the cells have variable expression of CD20 and CD22.^5^

A study of 123 canine lymphomas, not confined to the ocular region, found three cases of B‐LBL and eight cases of T‐LBL. These had densely stained cells in hematoxylin‐eosin staining of moderate size with intermediate‐sized nuclei and finely distributed chromatin, indistinct nucleoli, and scant cytoplasm.^3^ The architecture of the cells was diffuse, and there was a high mitotic rate.^3^

Canine lymphoma can be staged according to the World Health Organization staging system: stage I with involvement of a single node or a single organ (excluding bone marrow); stage II with regional involvement of multiple lymph nodes (± tonsils); stage III with generalized lymph node involvement; stage IV being stages I‐III with involvement of liver and/or spleen; and stage V being stages I‐IV with involvement of blood or bone marrow. Furthermore, canine lymphoma can be substaged with A (absence of systemic signs) or B (presence of systemic signs: fever, >10% weight loss, hypercalcemia).^6^

Periocular and intraocular presentations in lymphomas can be seen in dogs. However, these tumors have received limited attention in the literature.^7^, ^8^, ^9^, ^10^, ^11^, ^12^, ^13^, ^14^, ^15^, ^16^ To the best of our knowledge, this is the first case in the literature to report B‐LBL as the first presenting sign in the ocular region in a dog.

## 2 CASE REPORT

A 2‐year‐and‐7‐month‐old, intact, female Springer Spaniel presented to a veterinary ophthalmology referral clinic with a 1‐month history of a unilateral problem of the nictitating membrane. Sixteen days prior to referral, the patient was treated with an injection of amoxicillin trihydrate 150 mg/mL (Curamox Prolongatum^®^, Boehringer Ingelheim A/S, Copenhagen, Denmark), amoxicillin trihydrate and clavulanic acid 250 mg/12.5 mg (Clavubactin^®^, Dechra Veterinary Products A/S, Uldum, Denmark) a half tablet twice a day, and fusidic acid (Isathal®, Dechra Veterinary Products A/S) eye drops 10 mg/g in viscous vehicle one drop twice a day.

After 9 days, no improvement was observed and fusidic acid was discontinued. Topical dexamethasone sodium phosphate and chloramphenicol 1 mg/mL/5 mg/mL (Spersadex Comp^®^, Laboratoires THEA, Clermont‐Ferrand, France) was instilled one drop twice a day.

On presentation, a protrusion of the right side nictitating membrane (NM) was evident. On the bulbar aspect of the NM, the tumor area was thickened to approximately 5 mm, flabby, and mildly hyperemic. Slit‐lamp biomicroscopy (SL‐17, Kowa Ltd., Nagoya, Japan) of the cornea, anterior chamber, iris, and lens was unremarkable. Indirect ophthalmoscopy was not performed. Schirmer tear testing (STT, Mark Blu Optitech Eyecare, Allahabad, India) was 20 mm/min OD and 19 mm/min OS. Intraocular pressure measured with applanation tonometry (Tonopen Vet Medtronic Solan, Reichert Technologies, Munich, Germany) was 20 mm Hg OD and 17 mm Hg OS. Direct and indirect pupillary light reflex, menace response, and palpebral reflexes were normal. Examination of the oral cavity showed no abnormal signs. The weight was 17.6 kg and, apart from the eye problem, the patient was agile and in a good health condition. No laboratory tests were performed at this time. Local treatment from the referring veterinarian continued in this period.

Six days later, the general condition was unchanged; however, the thickness of the NM had increased to 15 mm. The patient was sedated with intramuscular 0.2 mg/kg methadone hydrochloride (Comfortan^®^ 10 mg/mL, Dechra Veterinary Products A/S), 2 µg/kg dexmedetomidine hydrochloride (Dexdomitor^®^ 0.1 mg/mL, Orion Pharma Animal Health, Copenhagen, Denmark), and 5.7 µg/kg acepromazine (Plegicil^®^ 10 mg/mL, Pharmaxin AB, Helsingborg, Sweden). After 20 minutes, the patient was induced with propofol (Propovet Multidose^®^ 10 mg/mL, Zoetis Finland OY, Helsinki, Finland) in a catheter through the saphenous vein of the right side until effect, in total 35 mL. After endotracheal intubation, the patient was maintained on isoflurane (Attane Vet^®^, ScanVet Animal Health, Fredensborg, Denmark) saturated in 100% oxygen.

A transpalpebral ultrasound scan with linear probe SL 1543 (Esaote MyLab Gamma, Genova, Italy) revealed no bulbar or retrobulbar involvement. A small amount of fluid with a high number of neutrophils was retrieved with fine needle aspiration from the NM swelling.

On suspicion of an abscess or intramembranal foreign body, the membrane was bluntly opened caudal to the T‐shaped cartilage. An amount of 0.5 to 1 mL pus‐like fluid with two or three small foreign bodies resembling plant material escaped. The cavity was flushed through a contralateral opening with a 0.9% NaCl solution. Openings were left open for secondary intention healing.

Topical chloramphenicol (Kloramfenikol Viskouse DAK^®^, Takeda Pharma A/S, Taastrup, Denmark) and carprofen 50 mg 4 mg/kg per oral (Norodyl Vet^®^, ScanVet Animal Health) continued postoperatively. Due to the initial suspicion of an abscess and the fact that the patient was young, no staging for lymphoma was done at this point.

Another 6 days later, the NM protruded even more, but the patient still showed no discomfort. The swelling had become more firm and multinodular with no content of pus. A small sample of tissue of the NM was harvested for histopathology under general anesthesia. Postoperative medication continued unchanged.

**Figure 1:**
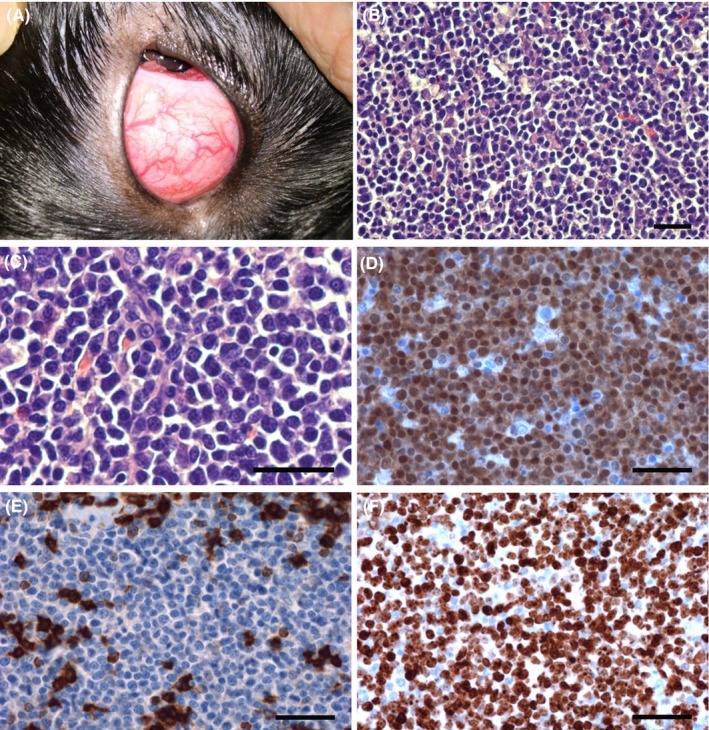
B‐cell lymphoblastic lymphoma of the nictitating membrane. Clinical and histological findings. A, Clinical appearance of the tumor of the nictitating membrane, showing a pinkish tumor measuring 3 × 2.5 × 1.5 cm. The tumor is well vascularized with several new tumor vessels; B, Microscopic overview showing diffuse monotonous infiltration of tumor cells (hematoxylin‐eosin, bar = 50 µm); C, The tumor cells were of medium size and showed pleomorphic features and blast morphology (hematoxylin‐eosin, bar = 50 µm); D, The tumor cells were positive for PAX5 (bar = 50 µm); E, were negative for CD3; however, scattered positive T cells were innocent bystanders (bar = 50 µm); F, Approximately 90% of the cells were positive for Ki‐67, demonstrating a high proliferation index of the tumor cells (bar = 50 µm)

The NM continued to enlarge for 2 weeks and started to cause the patient discomfort (Figure 1A). A decision to remove the NM was made. After standard pre‐surgical procedure, the NM was lifted and a full resection performed with the openings left for secondary intention healing. Postsurgical treatment with chloramphenicol and carprofen continued. The tissue was submitted for histopathological investigation.

At follow‐up 37 days after initial presentation, the eye was comfortable and the wound in the conjunctiva was healing properly. However, the general condition had deteriorated and the patient was now in poor condition. During the last days, the patient developed inappetence and depression, with moderate weight loss, a high temperature of 39.3°C, and generalized lymphadenopathy. While awaiting the result of the histopathology, a treatment with subcutaneously administered steroids against a suspected lymphoma was initiated with dexamethasone sodium phosphate 0.1 mg/kg (Rapidexon^®^ 2 mg/mL, Dechra Veterinary Products A/S). The diagnosis of a malignant lymphoma in the NM was reported. Due to the initial suspicion of an abscess and the fact the patient initially presented with no other signs of affection, no staging was done initially. We could not determine whether this was a primary lymphoma disseminating or a secondary lymphoma disseminated from elsewhere in the body, because of the lacking initial staging.

The owner had decided not to continue treatment in case of a malignant disease, and thus, no further staging was done after the suspicion of lymphoma arose. The patient was euthanized and, in accordance with the owner's wish, samples from the patient could be used for scientific purposes.

## 3 NECROPSY

**Figure 2:**
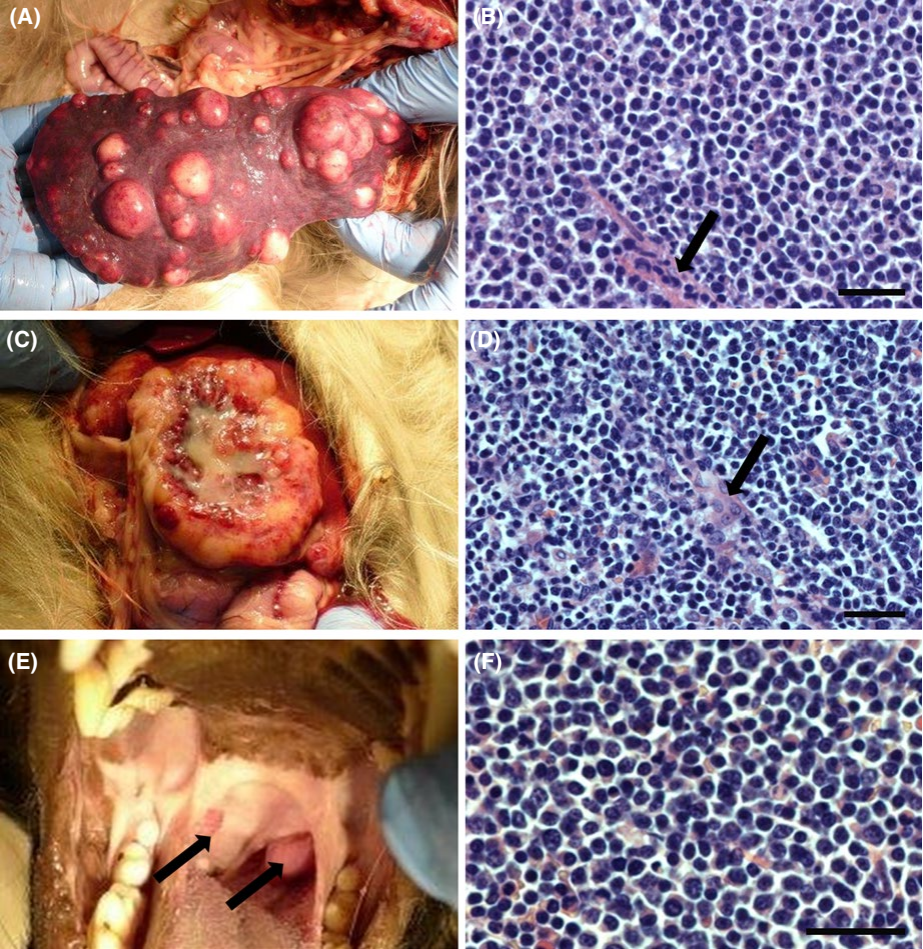
Necropsy observations demonstrating macroscopic and microscopic findings in different organs. A, The spleen was heavily infiltrated by numerous tumors ranging from a few millimeters to 3‐4 cm; B, The microscopic examination of the spleen showed diffuse infiltration of medium‐sized pleomorphic tumor cells. In the specimen, sinusoids (arrow) can be seen (hematoxylin‐eosin, bar = 50 µm); C, A horseshoe‐shaped process of 8 × 6 cm was showing in one liver lobe. The lesion showed central invagination with central necrosis and purulent secretion; D, The liver was diffusely infiltrated with several medium‐sized, pleomorphic tumor cells. Scattered hepatocytes (arrow) can be seen (hematoxylin‐eosin, bar = 50 µm); E, The tonsils of both sides were moderately enlarged (arrows); F, The tonsils were likewise heavily and diffusely infiltrated with tumor cells (hematoxylin‐eosin, bar = 50 µm)

Necropsy showed a generalized condition with tumor growth in the liver, spleen, and left ovary. Lymphadenopathy was pronounced throughout the whole body. Carcass weight was 16.5 kg, showing a mild weight reduction of 1.1 kg since initial presentation. In the spleen, disseminated growth of firm, round tumors ranging from a few millimeters to 3‐4 cm in diameter were found (Figure 2A). In one liver lobe, there was a horseshoe to umbilicated‐shaped process of 8 × 6 cm with central invagination and purulent secretion, suggesting tumor growth with severe necrosis in the center (Figure 2C). The left ovary measured 10 × 6 × 4 cm containing one large, firm homogenous tumor. Tonsillar lymph nodes were moderately enlarged (Figure 2E). The cranial sternal lymph node measured 4 × 3 × 2 cm. Especially in the abdomen, intestinal lymph nodes were conspicuous, with considerable thickening of the small intestinal walls.

## 4 MICROSCOPIC AND IMMUNOHISTOPATHOLOGICAL EXAMINATION

### 4.1 Materials and methods

All specimens were processed routinely, paraffin‐embedded, sectioned, and stained with hematoxylin‐eosin. Further staining with Giemsa‐AR was performed. Immunohistochemical procedures were performed on a Ventana Benchmark ULTRA platform (Ventana Medical Systems Inc., Tucson, AZ, USA) as previously described^17^ with antibodies CD3 (clone 2GV6, code 790‐4341, rabbit anti‐human, ready‐to‐use (RTU), Roche Diagnostics A/S, Hvidovre, Denmark), CD4 (clone SP35, code 790‐4423, rabbit anti‐human, RTU, Roche Diagnostics A/S), CD8 (clone SP57, code 790‐4460, rabbit anti‐human, RTU, Roche Diagnostics A/S), CD20 (clone L26, code 760‐4377, mouse anti‐human, RTU, Roche Diagnostics A/S), CD79α (clone SP18, code 790‐4432, rabbit anti‐human, RTU, Roche Diagnostics A/S), CD34 (QBEnd/10, code 790‐2927, mouse anti‐human, RTU, Roche Diagnostics A/S), PAX5 (clone SP34, code 790‐4420, rabbit anti‐human, RTU, Roche Diagnostics A/S), and TdT (clone SEN28, code NCL‐L‐Tdt‐339, mouse anti‐human, 1:100, Novocastra, Newcastle, UK). For each of the above‐mentioned antibodies, we ran both internal and external controls to validate our stainings. The external controls were human tissue, whereas we used a normal lymph node from the same patient as internal control. The positive external controls expressed the proteins of interest, whereas the negative external controls did not express the proteins of interest, indicating that the procedure was working. The lymph node demonstrated CD3+, CD4+, and CD8+ T cells in the parafollicular cortex (positive internal control), whereas no cells stained positive for these T‐cell markers in the follicles of the lymph node (negative internal control). Furthermore, a non‐specific negative control for each antibody was performed. These internal controls indicate that the stainings could be used in this particular patient. The specimens with tumor infiltration were also stained with Ki‐67 (clone MIB1, code M724001, mouse anti‐human, 1:100, Dako, Glostrup, Denmark).

## 5 RESULTS

### 5.1 The nictitating membrane

The tumor measured 3 × 2.5 × 1.5 cm. The tissue was made of connective tissue and fat tissue with isles of cartilage. The tissue was completely infiltrated by pleomorphic, non‐pigmented tumor cells. The tumor cells were of blast morphology. They were densely stained round cells of moderate size with intermediate‐sized nuclei. The cell borders were distinct. The chromatin was finely distributed with almost indistinct nucleoli. The cytoplasm was scant (Figure 1B,C). A starry‐sky configuration could be observed. There were 13 mitotic figures per high power field (×400 magnification). The tumor cells were infiltrating diffusely, and there was no necrosis. The tumor cells stained positive for PAX5 (Figure 1D) and negative for CD3 (Figure 1E) and CD34. The tumor cells did not stain for TdT or the T‐cell markers CD4 or CD8; nor did they stain for the B‐cell markers CD20 and CD79α. The Ki‐67 index was 90% (Figure 1F). The morphology and immunohistochemistry were consistent with the diagnosis of B‐LBL.

### 5.2 Necropsy

#### 5.2.1 Spleen

The white and red pulp was almost completely substituted by B‐LBL tumor cells (Figure 2B). These cells were positive for PAX5 and negative for CD3 and CD34. The Ki‐67 proliferation index was 40%‐50%.

#### 5.2.2 Liver

Several lobules were completely infiltrated with tumor cells of B‐LBL (Figure 2D). The cells were PAX5+, CD3−, and CD34−. The Ki‐67 proliferation index was 40%‐50%.

#### 5.2.3 Tonsils

The crypts of the tonsil were heavily and diffusely infiltrated with tumor cells of B‐LBL, making it difficult to see the normal tonsil structure of the specimen (Figure 2F). The tumor cells were positive for PAX5 and negative for CD3 and CD34. The Ki‐67 proliferation index was 50%‐60%.

#### 5.2.4 Popliteal lymph node

In the cortex of the lymph node, B cells were found in follicles. In the parafollicular cortex, CD3+ T cells, CD4+ T cells, and CD8+ T cells were found. The medulla contained lines of B cells in variable stages as well as macrophages. This specimen showed no signs of infiltration of tumor cells.

#### 5.2.5 Bone marrow

Blood vessels, hematopoietic cells, and adipocytes were diffusely infiltrated by B‐LBL tumor cells. The tumor cells demonstrated positivity for PAX5 and negativity for CD3 and CD34. The Ki‐67 proliferation index was 40%‐50%.

### 5.3 Staging

As described above, heavy tumor infiltration of several organs was present at necropsy. The tumor stage of our case was thereby stage VB with involvement of bone marrow and a systemic sign, fever.

## 6 DISCUSSION

We herein present a case of B‐LBL in a 2‐year‐and‐7‐month‐old, intact, female Springer Spaniel with the first presenting sign in the NM of a systemic disease. Lymphoblastic lymphomas are the most aggressive lymphoma subtypes encountered commonly in veterinary practice.^18^ Likewise, the present case showed a very aggressive behavior. LBL typically presents with the dog being visibly ill but in good condition, indicating a rapid onset of the lymphoma.^18^

Primary unicentric canine periocular and intraocular lymphomas are rare. However, ocular manifestations in multicentric lymphomas are quite common. Some reports suggest that one‐third of multicentric lymphomas have ocular involvement, making it the second most common clinical sign.^19^

Canine B‐LBL is less common than the T‐cell counterpart T‐LBL^3^; however, it seems that LBL is more frequent in younger individuals, as in the present case.^20^

When clinical suspicion of a lymphoma is raised, a thorough diagnostic procedure should begin. This includes clinical evaluation, computed tomography (CT) scan, and a bone marrow biopsy in order to determine the stage. Due to prognosis as well as treatment options, it is of great importance to know the specific subtype of lymphoma. By applying immunohistochemistry in canine lymphoma, it has become possible to make the specific diagnosis. In the present case, we saw diffuse infiltration of lymphoblastic cells; however, to further classify the lymphoma, we stained with the T‐cell markers CD3, CD4, CD8, and the B‐cell markers CD20, CD79α, as well as PAX5. We also stained for CD34, which can be used as a marker for the leukemic B‐cell subtype, B‐cell acute lymphoblastic leukemia. We used internal as well as external controls for each specimen and each stain. The positive internal controls indicate that our immunostainings did work in this particular patient and that the tumor cells were positive for PAX5 and negative for CD3, CD4, and CD8, and thus, the diagnosis of B‐LBL was confirmed.

The high Ki‐67 proliferation index of the tumor of the NM suggests a very aggressive behavior, and the rapid growth of the tumor likewise suggests aggressive behavior.

Lymphoblastic lymphoma is a highly aggressive subtype of lymphoma with a rapid clinical course. The B‐cell variant is less common than the T‐cell counterpart in canines, and little information about the clinical course is known for both subtypes. In a study of 13 cases of T‐LBL, the longest duration of clinical signs was 4 weeks and all patients were either stage III with generalized lymph node enlargement in both the front half and the back half of the body or stage IV with involvement of the liver and/or spleen, suggesting a very aggressive behavior.^20^ Due to the aggressive nature of B‐LBL and the widespread lymphoma, chemotherapy is the therapy of choice for lymphoblastic lymphoma, regardless of B‐cell or T‐cell linage.

To better understand lymphomas and their subtypes in the canine family, further research is needed. With the use of immunohistochemistry, it has become possible to diagnose lymphomas more specifically, and in the future, this can provide valuable information regarding lymphoma subtypes in the eye region. This will lead to more differentiated information regarding prognosis and available therapeutic modalities.

## AUTHOR CONTRIBUTIONS

FH, TH, and SH: involved in study conception and design. FH, TH, EC, and SH: performed acquisition of data. FH, TH, EC, LHM, and SH: performed analysis and interpretation of data. FH, TH, and SH: drafted the manuscript. FH, TH, EC, LHM, and SH: performed critical revision.

## References

[1] Dobson JM , Samuel S , Milstein H , et al. Canine neoplasia in the UK: estimates of incidence rates from a population of insured dogs. J Small Anim Pract. 2002;43:240‐246. 12074288 10.1111/j.1748-5827.2002.tb00066.x

[2] Vail DM , Young K . Hematopoietic tumors In: Withrow SJ, Vail DM, eds. Withrow and MacEwen's Small Animal Clinical Oncology. St. Louis, MO: Saunders Elsevier, 2007:699–784.

[3] Vezzali E , Parodi AL , Marcato PS , et al. Histopathologic classification of 171 cases of canine and feline non‐Hodgkin lymphoma according to the WHO. Vet Comp Oncol. 2010;8:38–49. 20230580 10.1111/j.1476-5829.2009.00201.x

[4] Valli VE , Kass PH , San Myint M , et al. Canine lymphomas: association of classification type, disease stage, tumor subtype, mitotic rate, and treatment with survival. Vet Pathol. 2013;50:738–748. 23444036 10.1177/0300985813478210

[5] Jaffe ES , Harris NL , Stein H , et al. World Health Organization Classification of Tumours. Pathology and Genetics of Tumours of Haematopoietic and Lymphoid Tissues. Lyon, France: IARC Press; 2001.

[6] Zandvliet M . Canine lymphoma: a review. Vet Q. 2016;36:76–104. 26953614 10.1080/01652176.2016.1152633

[7] Ota‐Kuroki J , Ragsdale JM , Bawa B , et al. Intraocular and periocular lymphoma in dogs and cats: a retrospective review of 21 cases (2001–2012). Vet Ophthalmol. 2014;17:389–396. 24118744 10.1111/vop.12106

[8] Donaldson D , Day MJ . Epitheliotropic lymphoma (mycosis fungoides) presenting as blepharoconjunctivitis in an Irish setter. J Small Anim Pract. 2000;41:317–320. 10976628 10.1111/j.1748-5827.2000.tb03209.x

[9] Olbertz L , Lima L , Langohr I , et al. Supposed primary conjunctival lymphoma in a dog. Vet Ophthalmol. 2013;16(Suppl. 1):100–104. 22524231 10.1111/j.1463-5224.2012.01027.x

[10] Hong IH , Bae SH , Lee SG , et al. Mucosa‐associated lymphoid tissue lymphoma of the third eyelid conjunctiva in a dog. Vet Ophthalmol. 2011;14:61–65. 21199281 10.1111/j.1463-5224.2010.00843.x

[11] Vascellari M , Multari D , Mutinelli F . Unicentric extranodal lymphoma of the upper eyelid conjunctiva in a dog. Vet Ophthalmol. 2005;8:67–70. 15644103 10.1111/j.1463-5224.2005.04053.x

[12] McCowan C , Malcolm J , Hurn S , et al. Conjunctival lymphoma: immunophenotype and outcome in five dogs and three cats. Vet Ophthalmol. 2014;17:351–357. 23910215 10.1111/vop.12083

[13] Escanilla N , Leiva M , Ordeix L , et al. Uveodermatologic lymphoma in two young related Portuguese water dogs. Vet Ophthalmol. 2012;15:345–350. 22239173 10.1111/j.1463-5224.2011.00981.x

[14] Aquino SM , Hamor RE , Valli VE , et al. Progression of an orbital T‐cell rich B‐cell lymphoma to a B‐cell lymphoma in a dog. Vet Pathol. 2000;37:465–469. 11055871 10.1354/vp.37-5-465

[15] Malmberg JL , Garcia T , Dubielzig RR , et al. Canine and feline retinal lymphoma: a retrospective review of 12 cases. Vet Ophthalmol. 2017;20:73–78. 26868476 10.1111/vop.12356

[16] Cello RM , Hutcherson B . Ocular changes in malignant lymphoma of dogs. Cornell Vet. 1962;52:492–523. 14019615

[17] Mikkelsen LH , Andreasen S , Melchior LC , et al. Genomic and immunohistochemical characterisation of a lacrimal gland oncocytoma and review of literature. Oncol Lett. 2017;14:4176–4182. 28943925 10.3892/ol.2017.6713 PMC5604129

[18] Valli VE , San Myint M , Barthel A , et al. Classification of canine malignant lymphomas according to the World Health Organization criteria. Vet Pathol. 2011;48:198–211. 20861499 10.1177/0300985810379428

[19] Krohne S , Henderson N , Richardson R , et al. Prevalence of ocular involvement in dogs with multicentric lymphoma: prospective evaluation of 94 cases. Vet Comp Ophthalmol. 1994;4:127–135.

[20] Ponce F , Magnol JP , Blavier A , et al. Clinical, morphological and immunological study of 13 cases of canine lymphoblastic lymphoma: comparison with the human entity. Comp Clin Path. 2003;12:75–83.

